# Evaluation of the Effects of the Sodium–Glucose Cotransporter 2 Inhibitors and Sacubitril/Valsartan Combined Therapy in Patients with HFrEF: An Echocardiographic Study

**DOI:** 10.3390/ijms26125651

**Published:** 2025-06-12

**Authors:** Isabella Fumarulo, Annalisa Pasquini, Giulia La Vecchia, Bianca Pellizzeri, Andriy Sten, Barbara Garramone, Marcello Vaccarella, Salvatore Emanuele Ravenna, Antonella Lombardo, Francesco Burzotta, Dario Pitocco, Nadia Aspromonte

**Affiliations:** 1 Department of Cardiovascular Sciences, Fondazione Policlinico Universitario A. Gemelli IRCCS, 00168 Rome, Italy; isabella.fumarulo@guest.policlinicogemelli.it (I.F.);; 2 Department of Cardiovascular Sciences, Catholic University of the Sacred Heart, 00168 Rome, Italy; 3 Operative Unit of Diagnostic Interventional Cardiology, Isola Tiberina-Gemelli Isola, 00186 Rome, Italy; 4 Division of Cardiology, Azienda Ospedaliero Universitaria Policlinico “G. Rodolico-San Marco”, University of Catania, 95100 Catania, Italy; 5 Department of Life Science, Health and Health Professions, Università degli Studi Link, 00165 Rome, Italy; 6 Diabetes Care Unit, Fondazione Policlinico Universitario A. Gemelli IRCCS, 00168 Rome, Italy

**Keywords:** heart failure, echocardiography, global longitudinal strain, gliflozins

## Abstract

Sodium–glucose cotransporter 2 inhibitors (iSGLT2) have become the fourth pillar of the medical treatment for heart failure with reduced ejection fraction (HFrEF). However, the mechanisms of action of iSGLT2 remain poorly understood. The effectiveness of combined ARNI and iSGLT2 therapy in left ventricular (LV) remodeling is still under study. We aim to investigate the effects of ARNI + iSGLT2 combination therapy in patients affected by HFrEF in terms of ventricular remodeling using speckle tracking echocardiography (STE). In this observational study, 136 patients with HFrEF taking ARNI were enrolled. All patients were evaluated at baseline (before iSGLT2), at 3 months and at 12 months from the beginning of iSGLT2 therapy. Echocardiographic parameters, including STE analysis and volumetric and LV contractile function indices, were collected at the three timepoints. The objectives were (1) to evaluate the effects of ARNI + iSGLT2 combination therapy on ultrasound (US) measurements; (2) to evaluate the effects on the variation of laboratory data indicative of HF (NT-pro-BNP); and (3) to evaluate the medium-long term impact of the ARNI + iSGLT2 combination therapy in terms of major cardiovascular events (MACVE). After only three months of combined ARNI + iSGLT2 therapy, we reported a significant improvement in ventricular and atrial volumetric indices, systolic function indices and myocardial deformation parameters assessed by STE. We also reported a significant decrease in NTproBNP levels. This trend was confirmed at 12 months follow-up. Furthermore, narrowing down the analysis to patients who were already treated with ARNI when they started taking iSGLT2, we reported similar results in the improvement of US parameters and NTproBNP levels. Our study has shown that the ARNI + iSGLT2 combination therapy leads to a clinical improvement and positive ventricular remodeling. Even the single introduction of additional iSGLT-2 in HFrEF patients on an otherwise optimized therapy resulted in a significant improvement in US and laboratory variables. The results of our study suggest implementing iSGLT-2 therapy as soon as possible, as the structural and functional cardiac improvements achieved by these drugs are achieved in the short term and maintained in the long term.

## 1. Introduction

### 1.1. Heart Failure and Diabetes

Heart failure (HF) is a serious condition with a significant impact in terms of mortality and morbidity worldwide [1,2]. It is not a single, specific entity; instead, it constitutes the final pathway of many cardiovascular diseases, from various etiologies [3,4,5]. Diabetic patients have shown a higher risk of developing heart failure, also due to the concentric remodeling of the left ventricle induced by diabetes, which is responsible for a type of heart failure independent of the presence of ischemic heart disease or underlying vascular disease, known as “diabetic cardiomyopathy” [6].

The diagnosis of HF is based on clinical evaluation, which includes anamnesis and physical examination (signs and symptoms), and through laboratory and instrumental investigations [7,8].

Therapy with ARNI (Sacubitril/Valsartan) has been shown to significantly reduce the mortality rate and rehospitalization for heart failure [9], with beneficial effects also in terms of reverse myocardial remodeling [10]. More recently, SGLT2 inhibitors (Gliflozins), oral antidiabetic drugs, have shown significant beneficial effects on the risk of recurrent cardiovascular events in patients with heart failure with or without diabetes [11], becoming part of the recommended pharmacological treatment according to the most recent guidelines on the treatment of heart failure with reduced ejection fraction [12]. Several studies have demonstrated the positive role of dapagliflozin [13] and empagliflozin [14] in heart failure, while ertugliflozin has also had promising results in recent trials [15]. However, whether therapy with SGLT2 inhibitors, in addition to therapy with ARNI, in patients with heart failure with reduced ejection fraction is effective in terms of reverse myocardial remodeling and reducing the risk of adverse cardiovascular events remains subject to study.

### 1.2. Literature Review

Several trials have analyzed the effects of ARNI and SGLT2i in patients with HFrEF, in terms of mortality and hospitalizations (PARADIGM-HF, EMPEROR-Reduced trial, DAPA-HF, TRANSITION-HF, PIONEER-HF). Only a few studies, however, have analyzed the quantifiable effects of the two drugs on patients’ hearts. A recent meta-analysis [16], in which 32 studies were collected with 2351 patients, showed that the use of SGLT2 inhibitors was associated with an improvement in LV contractile function but without a significant improvement in the LV end-diastolic volume. Likewise, subsequent studies [17,18] have demonstrated that the addition of an SGLT2 inhibitor to sacubitril/valsartan and other maximally tolerated baseline therapies in HFrEF resulted in greater improvement of LV systolic function compared with a treatment regimen without SGLT2i. Recently, Pastore et al. [19] demonstrated a beneficial effect of dapagliflozin in nondiabetic patients with HFrEF and HFmrEF in terms of myocardial performance measured by STE; however, a favorable effect on cardiac remodeling in volumetric terms has not yet been demonstrated on a large scale or considering treatment with both Dapagliflozin and Empagliflozin.

### 1.3. Heart Failure Therapy

HF therapy can rely on drugs, devices and, in the last stages, on replacement therapies [20,21]. The latest ESC 2021 guidelines on the treatment of heart failure with reduced ejection fraction recommend the introduction of therapy with ARNI and SGLT2 inhibitors as first-line treatment in patients with HFrEF, along with beta-blockers and potassium-sparing diuretics, with a Class I, Level of Evidence A recommendation [12]. ARNI and SGLT2 inhibitors, thus, become part of the “four pillars” of therapy for patients with HFrEF. However, the ESC guidelines do not specify the timing for introducing these four classes of drugs, which remains at the discretion of the prescribing physician.

### 1.4. Global Longitudinal Strain

In the last few years, GLS has been used as a predictive and prognostic factor in many different groups, including HF patients and in the general population [22], as well as cardio-oncology [23] and amyloidosis [24]. In patients with diabetes, the GLS has been demonstrated to have a strong prognostic significance in predicting new-onset HF [25]. GLS might also be useful in predicting the efficacy of interventional treatment in patients with HFrEF, with worse outcomes in diabetic patients [26].

## 2. Results

One hundred thirty-six patients have been enrolled in our study. One hundred fourteen (83.8%) were males and 22 (16.2%) females, with an average age of 64.3 ± 12.5 years and an average eGFR of 69.7 mL/min ± 24.6 mL/min. Almost all of the patients (99%) were under beta-blocking therapy, and since the study analyzed the effects of SGLT2i, every patient (100%) was taking SGLT2i. Every patient (100%) was taking ARNI, for the same reason mentioned above, at the most effective prescribable doses: Sac/Val 24/26 mg (28.2%); Sac/Val 49/51 mg (21.8%); and Sac/Val 97/103 mg (50%). Specifically, 65% of the patients were already on ARNI before t0, while the remaining 35% started ARNI therapy at t0. Of these patients, 31% were previously taking an RAA medication (ACE inhibitor or ARB), while 69% were not. ARNI up-titration occurred in 19% of patients during follow-up. MRA (77.3%), diuretics (61.8%), and anticoagulants (50.9%) were the other three most prevalent medications our group of patients was taking. Diuretics were reduced in 27% of patients during follow-up due to clinical improvement. With regards to CV risk factors, the most present were hypertension (68.2%), dyslipidemia (64.5%), and previous smoking (58.2%). Other less prevalent comorbidities were familiarity for CVD (29.1%), DM (14.5%), and active smoking (9.1%). The most prevalent comorbidities in our group of patients were CKD (45.4%) and history of AFib (44.5%). Other important comorbidities to consider, such as COPD (26.4%), peripheral arterial disease (20.9%), and distiroidism (17.2%), have also been considered. Some of the less present comorbidities include previous TIA/Stroke (9.1%), chronic anemia (2.7%), and liver disease (1.8%). The vast majority of our 136 patient group was included in two main diagnoses: primitive/idiopathic DCM (43%) and ischemic cardiomyopathy (35%). The remaining 22% of the diagnoses were divided between sequelae of myocarditis (7%), valvular heart disease (6%), non-compacted myocardium (5%), chemotoxicity (2%) and other (2%) (Table 1).

For each timepoint analysis (baseline, 3 months and 12 months), only patients with complete echocardiographic and laboratory data available at both timepoints were included. Therefore, there were minor differences in the number of patients contributing to baseline values across comparisons (t0–t1 vs. t0–t2), and, consequently, slight discrepancies in the reported baseline means. The baseline values presented in comparative analyses refer to the respective subgroups with full paired data.

**Figure 1:**
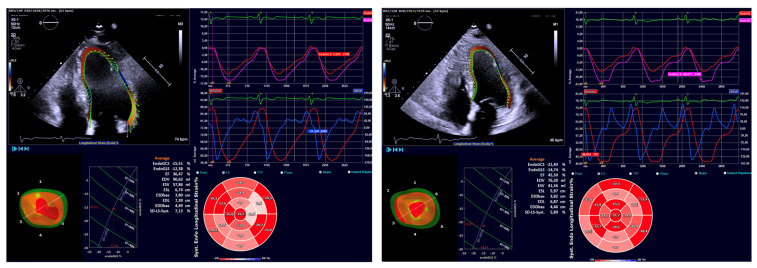
Changes in myocardial deformation parameters of LV assessed by STE after 3 months of iSGLT2 therapy. Baseline (t0) on the left; 3 months (t1) on the right. At each time point: (1) Top left: endocardial tracking; (2) bottom left: GCS; (3) bottom right: GLS; (4) top right: evolution over time of endomyocardial deformation (top) and volumes (bottom). GCS: global circumferential strain; GLS: global longitudinal strain; LV: left ventricle; STE: speckle tracking echocardiography.

After three months of combined ARNI and iSGLT2 therapy, significant improvements were observed in ventricular and atrial volumetric indices (LVEDVi 87.8 vs. 95.3 mL/m^2^; LVEDD 54.7 vs. 58.8 mm; LAVi 46.5 vs. 49.2 mL/m^2^, each with a *p*-value < 0.05), systolic function indices of the left ventricle (LVEF 39% vs. 31%, *p*-value < 0.05), and myocardial deformation parameters of LV assessed by STE (LVGLS −11.2% vs. −8.9%, *p*-value < 0.05) (Figure 1) (Table 2).

Likewise, diastolic function improved (grade 1.3 vs. 1.7, *p*-value < 0.05). A slight improvement in RV systolic function and in estimated pulmonary artery systolic pressure seemed to be present; however, it was not statistically significant (TAPSE 18.8 vs. 18.4 mm, *p*-value 0.17; PAPs 34.3 vs. 36.7 mmHg, *p*-value 0.067). We also reported a tiny, still statistically significant improvement in the grade of mitral regurgitation (MR 0.9 vs. 1.1, *p*-value < 0.05), while other valvulopathies did not change significantly (TR 0.6 vs. 0.7, *p*-value 0.08).

**Figure 2:**
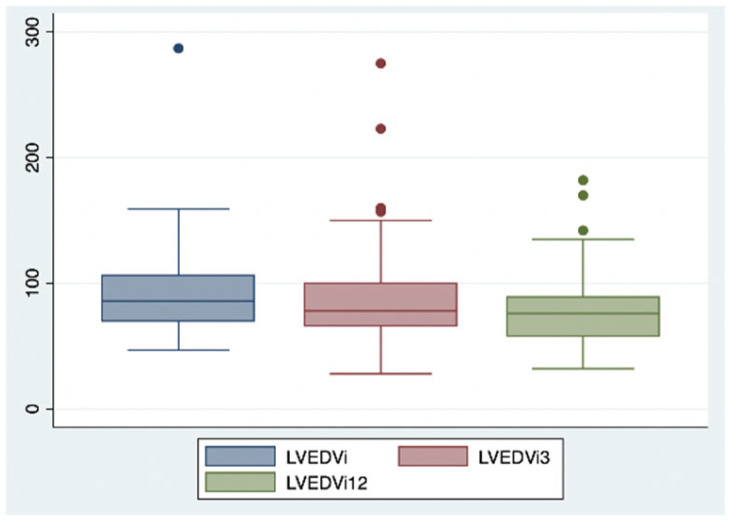
Changes in left ventricle end diastolic volume index after 3 and 12 months of iSGLT2 therapy.

**Figure 3:**
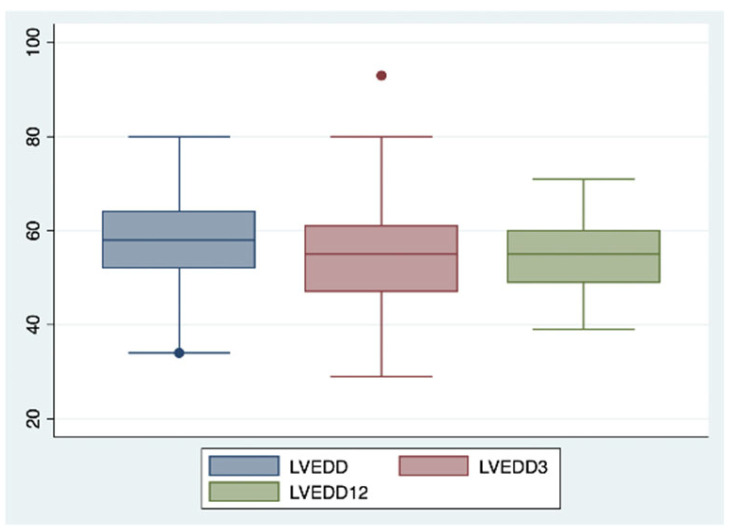
Changes in left ventricle end diastolic diameter after 3 and 12 months of iSGLT2 therapy.

**Figure 4:**
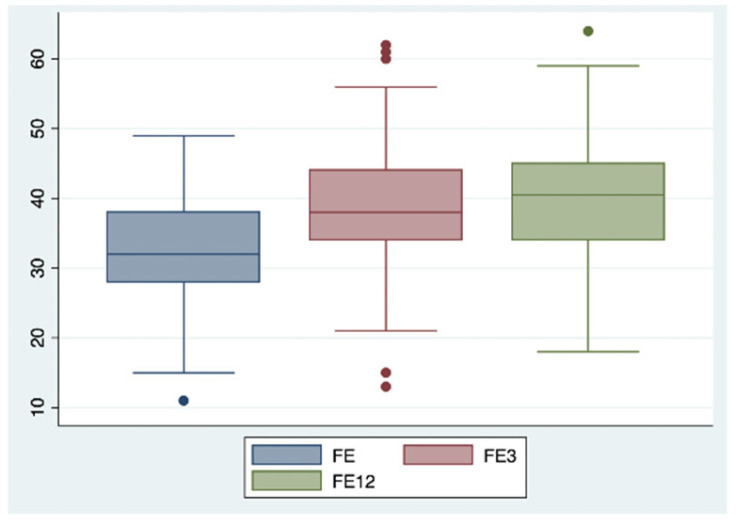
Changes in left ventricle ejection fraction after 3 and 12 months of iSGLT2 therapy.

**Figure 5:**
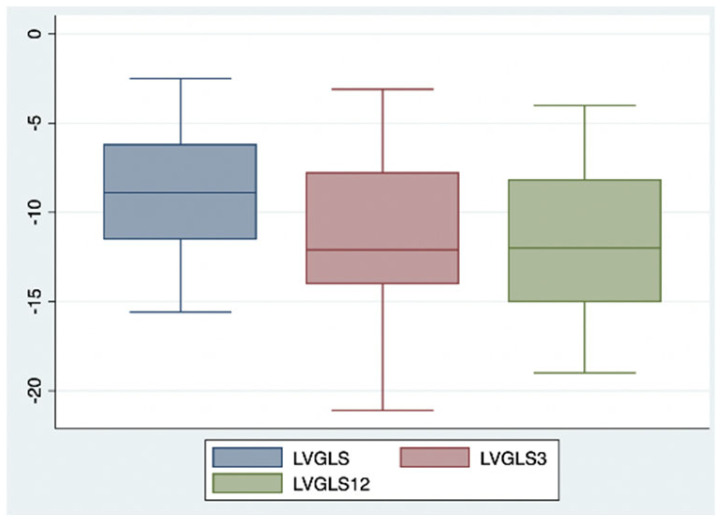
Changes in left ventricle global longitudinal strain after 3 and 12 months of iSGLT2 therapy.

This positive trend was confirmed after 12 months of combined therapy, with similar results observed in ventricular and atrial volumetric indices (LVEDVi 78.9 vs. 92.4 mL/m^2^; LVEDD 54.7 vs. 59.6 mm; LAVi 43.6 vs. 48.3 mL/m^2^, each with a *p*-value < 0.05) (Figure 2 and Figure 3), systolic function indices of the left ventricle (LVEF 40% vs. 31%, *p*-value < 0.05) (Figure 4), myocardial deformation parameters of LV assessed by STE (LVGLS −11.9% vs. −9.3%, *p*-value < 0.05) (Figure 5) and diastolic function (grade 1.3 vs. 1.6, *p*-value < 0.05).

Notably, the improvements in RV systolic function and in estimating pulmonary artery systolic pressure, only suspected at three months follow-up, became statistically significant at 12 months follow-up (TAPSE 19.6 vs. 18.3 mm; PAPs 31.6 vs. 36.5 mmHg, each with a *p*-value < 0.05).

A significant decrease in NT-proBNP levels was also reported after three months of combined ARNI and iSGLT2 therapy (1276 vs. 2411 pg/mL, *p*-value < 0.05), confirmed after 12 months (1029 vs. 2320 pg/mL, *p*-value < 0.05). In the subgroup of patients who underwent ARNI up-titration during follow-up (19% of patients), NT-proBNP decreased from a baseline median of 2602 pg/mL to 1086 pg/mL at 12 months, compared to a reduction from 2303 pg/mL to 1035 pg/mL in patients without up-titration. Although the difference did not reach statistical significance (*p*-value = 0.13), it suggests a possible trend toward a greater biomarker response in patients who tolerated up-titration. No significant differences were observed in LVEF or GLS changes between groups.

Furthermore, we conducted a subanalysis of patients already on ARNI who started taking iSGLT-2 (65% of the total). After three months of additional iSGLT2 treatment, we reported interesting but not statistically significant improvements in volumetric indices of LV (LVEDVi 89.9 vs. 92.7 mL/m^2^, *p*-value 0.14; LVEDD 55.9 vs. 57.7 mm, *p*-value 0.1), which became statistically significant at 12 months follow-up (LVEDVi 84.1 vs. 90.3 mL/m^2^; LVEDD 56 vs. 59.2 mm, each with *p*-value < 0.05). LV systolic function indices and myocardial deformation parameters assessed by STE both significantly improved already at three months follow-up (LVEF 37.4% vs. 32.9%; LVGLS −10.6% vs. −9.4%, each with *p*-value < 0.05), confirmed after 12 months (LVEF 38.5% vs. 32.8%; LVGLS −11% vs. −9.4%, each with *p*-value < 0.05). No significant improvements were reported in left atrial volumetric indices, diastolic function, RV systolic indices, or severity of valvulopathies, while a significant reduction in estimated pulmonary artery systolic pressure was documented at 12 months follow-up (PAPs 32.3 vs. 34.6 mmHg, *p*-value < 0.05). A significant decrease in NTproBNP levels was likewise reported at three months follow-up (1468.9 vs. 2135.2 pg/mL, *p*-value < 0.05) and confirmed at 12 months (1205.6 vs. 1964.5 pg/mL, *p*-value < 0.05).

We also conducted a subanalysis considering the diabetic status. Of the total patients, 14.5% were affected by diabetes mellitus (DM). Echocardiographic parameters improved similarly in both groups. In DM patients, LVEF increased from 31.5% to 35.9% at 3 months and to 38.1% at 12 months; in non-DM patients, from 30.7% to 36.4% at 3 months and to 39.2% at 12 months. GLS improved from −9.1% (t0) to −10.1% (t1) in the DM group and from −8.8% (t0) to −10.7% (t1) in the non-DM group at 3 months, continuing, respectively, to −10.9% (t3, DM group) and −11.4% (t3, non-DM group) at 12 months. LVEDVi and LVEDD also decreased in both groups, with a slightly greater reverse remodeling in non-diabetics. NT-proBNP decreased in both groups, although with a more pronounced decrease in non-DM patients (from 2454 pg/mL at baseline to 957 pg/mL at 12 months, compared to a reduction from 2243 pg/mL to 1391 pg/mL in diabetic patients).

Finally, MACVE occurred in 13% of patients. We performed a linear regression analysis, in which we found a statistically significant association between left atrial volume index (LAVi) values at baseline (t0) and MACVE at 12 months (t2) (*p*-value 0.05, 95% CI −0.005–+0.45). Therefore, baseline LAVi has been identified as a predictor, due to its independent association with MACVE.

## 3. Discussion

Our study aims to address the knowledge gap regarding the combined impact of ARNI and iSGLT2 on ventricular remodeling, as assessed by volumetric indices and advanced speckle tracking echocardiography (STE), as well as the long-term effects on heart failure biomarkers (NT-proBNP) and major adverse cardiovascular events (MACVE). Previous clinical trials have demonstrated significant benefits in terms of survival in HFrEF patients treated with ARNI and SGLT2i. However, the combined effect of these agents, particularly on the aspects of cardiac remodeling captured by STE, has been less well defined. This study provides interesting data on this topic.

We observed early (3 months) and sustained (12 months) significant improvements in ventricular and atrial volumetric indices (LVEDV, LVEDD, FE%), systolic function, and myocardial deformation parameters assessed by STE. Although our study did not include a control group treated with either ARNI or SGLT2i alone, the progressive improvement in structural and functional cardiac parameters observed in our patients from baseline to 3 and 12 months suggests that the synergistic or additive mechanisms of action of ARNI and SGLT2i may lead to a stronger and more rapid beneficial impact on cardiac remodeling and function improvement compared to either therapy alone. The mechanical effects of ARNI therapy include reducing wall stress, inhibiting myocardial fibrosis, and modulating neurohormonal pathways through neprilysin inhibition and RAAS blockade, as demonstrated in the PROVE-HF and EVALUATE-HF studies [27,28]. These effects finally promote myocardial reverse remodeling. On the other hand, SGLT2 inhibitors exert pleiotropic effects, at least in part metabolic, including natriuresis, reduction of interstitial volume, improvement in myocardial energetics, anti-inflammatory actions, and attenuation of negative remodeling, regardless of glycemic status [29,30].

The subgroup analysis showed that initiation of SGLT2i therapy in patients previously treated with ARNI still produced significant improvements, similar to those seen in patients who started ARNI and SGLT2i therapy concurrently. This was particularly evident for cardiac contractility parameters (LVEF and LVGLS), while there was a reported slight delay in volumetric remodeling in the subgroup with the addition of SGLT2i. This suggests that the benefits of adding an SGLT2i are evident even in patients already receiving the cornerstone HF therapy (ARNI + BB + MRA), reinforcing the guideline recommendations for broad SGLT2i use in HFrEF. Interestingly, NT-proBNP reduction was slightly more marked in non-diabetic patients, suggesting SGLT2i benefits extend beyond glycemic control, which is consistent with results from prior studies [14].

Finally, we found a statistically significant association between left atrial volume index (LAVi) values at baseline (t0) and MACVE at 12 months (t2). In other words, a larger atrium volume is predictive of worse outcomes in the long term. Therefore, baseline LAVi may help identify patients at higher risk of adverse outcomes. These data are consistent with the results of previous studies regarding various cardiovascular diseases [31,32,33,34], and highlight the importance of the left atrium evaluation in the overall assessment of the individual patient’s prognostic pathway.

However, as an observational study, it is important to acknowledge potential limitations. The retrospective component of the study introduces the possibility of selection bias and confounding factors. The lack of a control group receiving only ARNI or SGLT2i makes it challenging to definitively attribute the observed improvements solely to the combination therapy; therefore, a clear causal relationship cannot be established, though within-subject analysis and consistent trends across endpoints support a strong association. A small percentage of bias could have been generated by a concomitant titration of the rest of the medical therapy in those patients who required therapeutic adjustments. Actually, we performed a subgroup analysis of patients already on ARNI treatment. Nevertheless, a small percentage of these patients (19%) underwent a further up-titration of ARNI treatment during the study. In terms of demographics, only 16.2% of patients were female. This imbalance in selection was completely unintentional; however, it could cause a gender bias. Last, the dependence of STE on image quality and correct acquisition should be considered, although a high feasibility has been demonstrated in many studies [29,30].

Despite these limitations, the study provides interesting evidence supporting the early and sustained benefits of ARNI and SGLT2i combination therapy in HFrEF patients.

Future randomized controlled trials are needed to confirm these observational findings.

## 4. Materials and Methods

### 4.1. Study Design and Population

This observational study, both prospective and retrospective, included a total of 136 patients affected by heart failure with reduced ejection fraction (EF < 40%) undergoing therapy with Sacubitril/Valsartan and with an indication for initiating therapy with SGLT-2 inhibitors as per guidelines. The study has been approved by the Ethics Committee (protocol ID 5428, approved on date 9 February 2023). The enrollment phase began upon approval by the Ethics Committee and lasted 12 months. During this period, both patients who had not yet started iSGLT2 therapy (prospective arm) and patients who had started iSGLT2 therapy within the previous 12 months (retrospective arm) were enrolled. Therefore, we considered the baseline (t0) at the time of initiation of iSGLT2 therapy. The time of enrollment occurred at t0 in the prospective group, while in the retrospective group it occurred during the 12 months following t0. Additionally, we recorded whether the patient was already on ARNI therapy at t0 (and the respective dosage) or whether ARNI therapy was started at t0 together with iSGLT2 therapy. All the selected patients underwent, at the baseline (t0) and at approximately 3 and 12 months after the introduction of SGLT-2 inhibitor therapy (t1 and t2), clinical evaluation, echocardiography, and biomarker investigations to assess the medium to long-term clinical and echocardiographic impact of ARNI + SGLT-2 inhibitor combination therapy in terms of ventricular remodeling studied using speckle tracking echocardiography. Additionally, echocardiographic and laboratory parameters of heart failure that best predict the incidence of major cardiovascular events and medium to long-term myocardial remodeling in treated patients were evaluated. All procedures to which the patients were subjected (including the optimization of medical therapy) fall within the standard of care of normal clinical practice for the pathology considered, in accordance with the most recent guidelines; therefore, the study is substantially observational. For the same reason, since all patients were treated per the guidelines, the study lacks a control arm; rather, participant baseline parameters served as the control. During follow-up, we excluded patients who showed prohibitive intolerance (mainly hypotension for ARNI, urinary infections for iSGLT2, or reduction in renal function for both the drugs). The objectives of the study were as follows:

Primary endpoint:

- Assessing the mid-term (3 months) echocardiographic impact of the combination of ARNI + SGLT2 inhibitors in terms of ventricular remodeling, studied as variations in myocardial strain indices (GLS%) in patients with HFrEF receiving optimized medical therapy.

**Figure 6:**
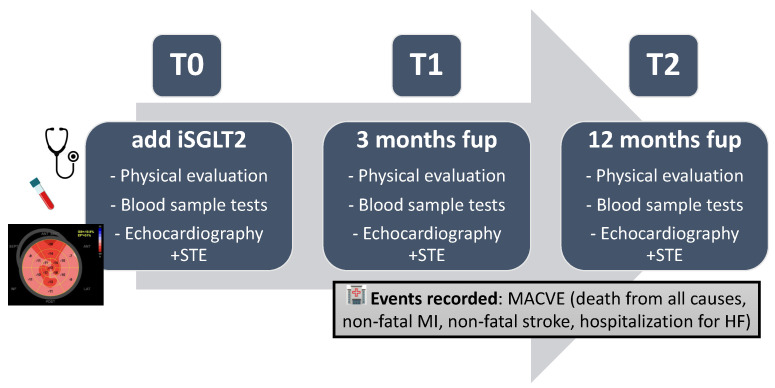
Central illustration. Fup: follow-up; HF: heart failure; iSGLT2: Sodium–glucose cotransporter 2 inhibitors; MACVE: major cardiovascular events; MI: myocardial infarction; STE: speckle tracking echocardiography.

Secondary Endpoints:

- Evaluate the long-term (12 months) echocardiographic impact of the ARNI + SGLT2 inhibitor combination in terms of ventricular remodeling, studied as variations in myocardial strain indices (GLS%) in patients with HFrEF receiving optimized medical therapy.
- Evaluate the clinical impact of the ARNI + SGLT2 inhibitor combination in terms of variation in laboratory data indicative of heart failure (NT-pro-BNP) in the mid-to-long term (3–12 months) in patients with HFrEF receiving optimized medical therapy.
- Evaluate the echocardiographic mid-to-long-term impact (3–12 months) of the ARNI + SGLT2 inhibitor combination on left ventricular remodeling, studied as variations in volumetric indices and contractile function (LVEDV, LVEDD, EF%) in patients with HFrEF receiving optimized medical therapy.
- Evaluate the clinical impact of the ARNI + SGLT2 inhibitor combination in terms of major adverse cardiovascular events (MACVE: composite endpoint of death from all causes, non-fatal myocardial infarction, non-fatal stroke and hospitalization for HF) in the mid-to-long term (3–12 months) in patients with HFrEF receiving optimized medical therapy.
- Identify echocardiographic parameters predictive of MACVE in the mid-to-long term (3–12 months) in patients with HFrEF after the introduction of combination therapy with ARNI + SGLT2 inhibitor.
- Identify laboratory parameters predictive of major adverse cardiovascular events (MACVE) in the mid-to-long term (3–12 months) in patients with HFrEF after the introduction of combination therapy with ARNI + SGLT2 inhibitor.
- Identify echocardiographic parameters predictive of ventricular remodeling (LVEDV, LVEDD, EF%) and variation in myocardial strain indices (GLS%) in the mid-to-long term (3–12 months) in patients with HFrEF after the introduction of combination therapy with ARNI + SGLT2 inhibitor.
- Identify laboratory parameters predictive of ventricular remodeling (LVEDV, LVEDD, EF%) and variation in myocardial strain indices (GLS%) in the mid-to-long term (3–12 months) in patients with HFrEF after the introduction of combination therapy with ARNI + SGLT2 inhibitor (Figure 6).

### 4.2. Echocardiographic Protocol

The echocardiographic protocol involves the use of the latest-generation echocardiographs (“GE HeathCare” Vivid E95 Ultra Edition systems, Chicago, IL, USA) and the off-line evaluation of myocardial deformation indices using the software QStrain Echo 4.0 (“Medis Medical Imaging”, Leiden, Paesi Bassi). The echocardiographic parameters were acquired in accordance with European guidelines [35]. The parameters taken into consideration concern both systolic and diastolic function, as well as indirect indices of cardiac chamber filling pressures: EDV (end-diastolic volume), ESV (end-systolic volume), EF (ejection fraction), EDD (end-diastolic diameter), ESD (end-systolic diameter), IVS (left ventricular hypertrophy), PW (left ventricular posterior wall thickness), LVMi (left ventricular mass index), LAV (left atrial volume), E/E’ (mitral flow velocity), diastolic function (expressed according to a numbering from 1 to 3, with increasing severity), TAPSE (tricuspid annulus systolic excursion), PAPs (systolic pulmonary pressure), S’ TDI RV (right ventricular tissue Doppler), LV GLS (left ventricular global longitudinal strain), RVLS (right ventricular free wall longitudinal strain), RV GLS (right ventricular global longitudinal strain), LA PALS (peak global longitudinal atrial strain), AS (aortic stenosis), MS (mitral stenosis), MR (mitral regurgitation), AR (aortic regurgitation), and TR (tricuspid regurgitation).

### 4.3. Statistical Analysis

Data analysis was conducted using STATA/SE version 14. The primary endpoint was the variation in global longitudinal strain (GLS%) at 3 months following initiation of SGLT2 inhibitor therapy. The expected effect size was small (Cohen’s d = 0.26), based on preliminary institutional data and literature showing modest but consistent improvements in GLS with optimized heart failure therapy [36]. The sample size was calculated based on this expected small effect size (Cohen’s d = 0.26), assuming an 80% power and a two-sided 95% confidence level. Based on this, a total sample size of 100 patients (50 enrolled prospectively over one year and 50 retrospectively from the prior year) was deemed adequate. Our study included 136 patients; therefore, the sample appears adequately powered to detect a significant association. Continuous variables were summarized using average values and standard deviation, while categorical variables were presented as frequencies and percentages. Patient characteristics were compared using the Mann-Whitney U test for continuous variables and the chi-square or Fisher’s exact tests for categorical variables. We investigated the relationship between echo, clinical and laboratory data at baseline and after 3 and 12 months of iSGLT2 therapy using paired tests for within-subject comparisons. A *p*-value less than 0.05 was considered statistically significant. Multivariate analyses included adjustments for age, sex, comorbidities, and risk factors.

## 5. Conclusions

Our study demonstrates that the combination therapy with iSGLT-2 + ARNI results in notable improvements in ventricular remodeling and laboratory indices of HF, regardless of whether the patient is affected by diabetes. Even the introduction of additional SGLT-2 inhibitors in HFrEF patients otherwise on optimized therapy led to significant enhancements in LV’s volumetric, systolic function indices, and myocardial deformation parameters after only three months of treatment. These improvements are maintained at 12 months follow-up, suggesting a long-term positive remodeling in cardiac chambers.

The results of our study suggest implementing iSGLT-2 therapy as soon as possible, as its effects are synergic and additive with the effects of ARNI therapy. The structural and functional cardiac improvements achieved by these two drugs are early and maintained in the long term.

We also found that LAVi has a prognostic value in this population, highlighting the importance of the left atrium evaluation in all HFrEF patients, as it may serve as a predictor of adverse cardiovascular outcomes.

## Figures and Tables

**Table 1:** Characteristics of the patients enrolled at baseline.

**Age (y/o), mean ± sd**	64.3 ± 12.5
**eGFR (mL/min), mean ± SD**	69.7 ± 24.6
**Sex**	
Male	83.8%
Female	16.2%
**Prevalence of medical therapy**	
B-blockers	99%
MRA	77.3%
iSGLT2	100%
Sac/Val 24/26 mg bid	28.2%
Sac/Val 49/51 mg bid	21.8%
Sac/Val 97/103 mg bid	50%
Other diuretics	61.8%
Anticoagulant	50.9%
**Cardiovascular risk factors**	
Diabetes mellitus	14.5%
Dyslipidemia	64.5%
Hypertension	68.2%
Active smoking	9.1%
Previous smoking	58.2%
Familiarity for CVD	29.1%
**Comorbidities**	
CKD	45.4%
History of AF	44.5%
Previous TIA/stroke	9.1%
Periferal arterial disease	20.9%
Liver disease	1.8%
Chronic anemia	2.7%
Distiroidism	17.2%
COPD	26.4%
**Cardiac diagnosis**	
primitive/idiopathic DCM	43%
ischemic cardiomyopathy	35%
myocarditis	7%
valvular heart disease	6%
non-compacted myocardium	5%
chemotoxicity	2%
others	2%

**Table 2:** Summary of echocardiographic and laboratory parameters (baseline, 3 months, 12 months). In bold, the parameters that reached statistical significance (*p*-value < 0.05).

Parameter	Baseline (T0)	3 Months (T1)	12 Months (T2)
LVEDVi (mL/m^2^)	95.3	**87.8**	**78.9**
LVEDD (mm)	58.8	**54.7**	**54.7**
LAVi (mL/m^2^)	49.2	**46.5**	**43.6**
LVEF (%)	31	**39**	**40**
LVGLS (%)	−8.9	−**11.2**	−**11.9**
Diastolic Function Grade	1.7	**1.3**	**1.3**
TAPSE (mm)	18.4	18.8	**19.6**
PAPs (mmHg)	36.7	34.3	**31.6**
MR Grade	1.1	**0.9**	**0.9**
TR Grade	0.7	0.6	0.6
NT-proBNP (pg/mL)	2411	**1276**	**1029**

## Data Availability

The data presented in this study are available on request from the corresponding author. The data are not publicly available due to privacy restrictions.
